# The Production of the Oral Mucosa of Antiendomysial and Anti—Tissue-Transglutaminase Antibodies in Patients with Celiac Disease: A Review

**DOI:** 10.1100/tsw.2010.228

**Published:** 2010-12-14

**Authors:** Domenico Compilato, Giuseppina Campisi, Luca Pastore, Antonio Carroccio

**Affiliations:** ^1^ Department of Oral Sciences, “G. Messina”, Section of Oral Medicine, University Hospital of Palermo, Italy; ^2^ Department of Surgical Sciences, University of Foggia, Italy; ^3^ Internal Medicine, Ospedali Riuniti di Sciacca, Italy

**Keywords:** celiac disease, oral mucosa, anti–tissue transglutaminase antibodies, antiendomysial antibodies, oral biopsy

## Abstract

Celiac disease (CD) is a lifelong, T cell—mediated enteropathy, triggered by the ingestion of gluten and related prolamins in genetically susceptible subjects, resulting in minor intestinal mucosal injury, including villous atrophy with crypt hyperplasia and intraepithelial lymphocytosis, and subsequent nutrient malabsorption. Although serological tests for antiendomysial (EMA) and anti—tissue transglutaminase (anti-tTG) autoantibodies are used to screen and follow up on patients with CD, diagnostic confirmation is still based on the histological examination of the small intestinal mucosa. Although the small intestinal mucosa is the main site of the gut involved in CD, other mucosal surfaces (such as gastric, rectal, ileal, and esophageal) belonging to the gastrointestinal tract and the gut-associated lymphoid tissue (GALT) can also be involved. A site that could be studied less invasively is the mouth, as it is the first part of the gastrointestinal system and a part of the GALT. Indeed, not only have various oral ailments been reported as possible atypical aspects of CD, but it has been also demonstrated that inflammatory changes occur after oral supramucosal application and a submucosal injection of gliadin into the oral mucosa of CD patients. However, to date, only two studies have assessed the capacity of the oral mucosa of untreated CD patients to EMA and anti-tTG antibodies. In this paper, we will review studies that evaluate the capacity of the oral mucosa to produce specific CD autoantibodies. Discrepancies in sensitivity from the two studies have revealed that biopsy is still not an adequate procedure for the routine diagnostic purposes of CD patients, and a more in-depth evaluation on a larger sample size with standardized collection and analysis methods is merited. However, the demonstration of immunological reactivity to the gluten ingestion of the oral mucosa of CD, in terms of IgA EMA and anti-tTG production, needs to be further evaluated in order to verify whether the oral mucosa is colonized by lymphocytes activated in the intestine or if gluten could stimulate naïve lymphocytes directly in the oral mucosa. This would have important implications for the pathogenesis, diagnosis, and treatment of CD.

## CD DEFINITION, EPIDEMIOLOGY, AND CLINICAL PRESENTATION

Celiac disease (CD), also known as celiac sprue or gluten-sensitive enteropathy (GSE), is a lifelong, T cell–mediated enteropathy, occurring in genetically susceptible individuals. It is triggered by the ingestion of gluten and related prolamins, plant storage proteins found in wheat, barley (hordeins), and rye (secalins)[1,2]. The disease is considered an autoimmune disorder nowadays, triggered by gluten ingestion but, once described in the literature, it was identified as a small intestinal mucosal injury, including villous atrophy with crypt hyperplasia and an intraepithelial lymphocytosis, numerical increase of plasma cells in the *lamina propria,* and subsequent nutrient malabsorption. The major predisposing genes are the histocompatibility leukocyte antigen (HLA)-DQ2 and DQ8 genotypes found in at least 95% of patients[3], although non-HLA genes have been recently reported to be involved in the pathogenesis of CD[4,5].

Originally thought to occur only rarely in childhood, CD is now recognized as a common condition that can be diagnosed at any age[1]. The estimated prevalence of CD in the general population of North America and Western Europe is approximately 1%, with a reasonable range of 0.71 to 1.25%[6]. The disease is more common in females than in males, with a female to male ratio generally accepted to be 2–2.1:1, although some have suggested it may be more equal[7].

The advances made in the last decade in our understanding of CD have revealed the disease to possess a heterogeneous, wide, and often unsuspected range of clinical presentations. The “typical” or “classic” form of CD, characterized by a malabsorption syndrome (i.e., chronic diarrhea, abdominal pain and distension, growth retardation, or weight loss) is less common. The majority of patients have “atypical” CD, with few or no gastrointestinal symptoms and a predominance of extraintestinal manifestations, such as a reduced stature, osteoporosis, iron deficiency, anemia, and abnormalities in liver function tests[8]. Furthermore, silent forms have also been described[1]. CD patients are prone to develop long-term complications (e.g., autoimmune diseases, malignancies)[9], which are responsible for a higher mortality rate than in the general population[10,11,12]. This fact justifies gluten withdrawal (currently the only effective treatment for CD), even in persons without gastrointestinal symptoms, since a gluten-free diet (GFD) seems to be protective against long-term complications[9].

## CD DIAGNOSIS

The diagnosis of CD is based on three key parameters: (1) case identification, (2) serological tests, and (3) a histology evaluation of a small intestinal biopsy. As previously mentioned, case identification can be difficult due to the high frequency of atypical or silent clinical presentation type, and physicians should be particularly suspect of CD in cases with no gastrointestinal symptoms.

Used as initial, noninvasive screening, serological testing is the first step in ascertaining a diagnosis of CD. The most sensitive and specific tests are the IgA class anti–tissue transglutaminase (anti-tTG) or antiendomysial antibodies (EMA). The IgA anti-tTG has 91–100% specificity and approximately 77–100% sensitivity. Serum IgA EMA is considered more specific with virtually 100% specificity, although a somewhat lower sensitivity than anti-tTG[13]. However, reports exist of lower sensitivity and specificity in the clinical practice setting[14,15,16]. In active CD, the presence of symptoms and high serum levels of EMA and anti-tTG antibodies, and their disappearance after a GFD, can be considered as confirming diagnosis[17]. Despite the high diagnostic accuracy of serological tests, the current diagnostic criteria for CD should not be based on symptoms displayed by the patients and/or findings in serological testing alone[18,19], and a small intestinal biopsy sampling is considered to be the gold standard.

However, aspects relating to duodenal histology also pose increasing problems in diagnosing CD. It is often the case that fully symptomatic CD patients present a duodenal histology that does not reveal villi atrophy, often considered the hallmark of CD. And in many cases, the duodenal histology findings are characterized by the infiltration of intraepithelial lymphocytes with minimal villi damage. This aspect must be considered unspecific and not indicative of CD as it can be due to several causes, including drug consumption (i.e., nonsteroidal anti-inflammatory drugs).

## THE PRODUCTION OF CD-SPECIFIC ANTIBODIES

There is recent evidence that the diagnostic accuracy of an intestinal biopsy can be greatly enhanced by measuring EMA and anti-tTG produced *in loco* by cultured duodenal mucosa biopsies from patients with active CD or from those in remission after *in vitro* stimulation with a peptic-tryptic digest of gliadin, which usually contains the major immune stimulatory epitopes[20,21,22,23]. Indeed, Picarelli and colleagues established that the small intestine of CD patients is the site of EMA production and that a gliadin challenge could induce their release[24]. Furthermore, they demonstrated that EMAs were not found in the culture supernatants of the biopsy samples of 18 controls, whereas these antibodies were detected in the culture supernatants of all 16 untreated CD patients. EMAs were not detected in the supernatants of biopsy samples cultured in medium only from 23 treated CD patients, but they were detected in 17 of the 23 biopsy samples challenged with gliadin.

## ROLE OF THE MOUTH AND ORAL MUCOSA IN CD STUDIES

Although the small intestinal mucosa is the main site of the gut's involvement in CD, other mucosal surfaces (such as oral, gastric, rectal, terminal ileum, and esophageal) belonging to the gastrointestinal tract and gut-associated lymphoid tissue (GALT), can also be involved[25,26,27,28,29,30,31,32,33,34,35,36,37,38,39,40]. Gluten-driven T-cell activation can be demonstrated in all these sites. The simplest site in which to conduct studies is the mouth. As the first part of the gastrointestinal tract, the oral cavity is the route for all ingested material, including products derived from cereals. It might be suspected that the oral cavity would react to ingested gluten in a similar manner to gut mucosa, revealing oral changes when the disease is untreated and an improvement in these changes when CD is treated with a GFD.

Indeed, not only have various oral manifestations been reported as possible atypical aspects of CD, but it has also been shown that inflammatory changes occur after oral supramucosal application and a submucosal injection of gliadin into the oral mucosa of GFD-treated CD patients[41,42,43]. Furthermore, data relating to the detection of anti-tTG antibodies in the saliva of patients with CD have confirmed the possibility that the oral cavity might be actively and immunologically involved in CD, and thus used as a new and less invasive site for an initial diagnosis of CD[44,45,46].

The oral mucosa is very often involved in CD, also from a histomorphological point of view, but histological findings are controversial and occasionally difficult to interpret. Differences in the histological features of oral mucosa were demonstrated in a study that involved subjects with treated CD (on a GFD), subjects with newly diagnosed CD, and healthy controls[43,47]. Specifically, CD patients on a GFD demonstrated a higher inflammatory infiltrate in the epithelium and the *lamina propria* as compared with untreated CD subjects and healthy control individuals.

These results have been confirmed by a recent study[48] in which no differences were observed in CD3+ count or B- and T-lymphocyte counts, either in the epithelium or the *lamina propria* of the clinically healthy oral mucosa in untreated CD patients and in controls. This seemed to confirm that the oral mucosa does not reflect the intestinal damage of CD. However, these authors have demonstrated that the oral mucosa was unexpectedly affected by spongiosis, probably linked to a local toxic effect which, in turn, was induced by gluten.

On the basis of the above histological findings, it could be concluded that the oral mucosa cannot be considered to be the counterpart of intestinal mucosa, at least in terms of lymphocytic infiltrate. Hence, it cannot be used for an initial diagnosis of CD since, as regards oral mucosal infiltration, persons with untreated CD do not differ from healthy control individuals. However, the previous observation of consistent inflammatory infiltration of the oral mucosa of CD patients on a GFD led us to hypothesize that an immunological “memory” of gluten hypersensitivity can be created. According to this hypothesis, the same authors[48] evaluated the reaction of oral mucosa to a local gluten challenge[41,42]. They demonstrated that oral supramucosal application and a submucosal injection of gliadin powder or gliadin peptides into patients with treated CD induced significant immunological changes, displayed by an increase in intraepithelial CD4+ T cells, as well as CD4+ and CD8+ T cells in the *lamina propria*. In conjunction with the evidence of anti-tTG detection in the saliva of CD patients, the above data suggest an immunological reactivity of the oral mucosa in patients with CD, and thus the possibility of using the oral mucosa as an alternative site by which to study the immunological changes potentially linked to CD. Despite the well-demonstrated immunological reactivity of the oral mucosa in patients with CD, only two preliminary recent studies[49,50] have assessed the ability of the oral mucosa of untreated CD patients to produce EMA and anti-tTG autoantibodies.

## ORAL BIOPSY PROCEDURE

Oral biopsy is a procedure that consists of taking vital tissue samples for histomorphological assessment as well as for laboratory/experimental studies as tissue cultures. There are some related contraindications (i.e., severely compromised state of health; presence of anatomical structures of vital importance, like vessels and nerves, near the biopsy site) and some absolute contraindications (i.e., potential dissemination of neoplastic cells; promotion of cell transformation), which must be taken into account when planning an oral biopsy. The procedure is relatively safe, whereas surgical risks linked to oral biopsy may include hemorrhage, infections, and creation of reactive scarring processes and lesions of the small nerve endings with consequent short- or long-term sensory loss.

There are two types of oral biopsy: (1) incisional, in which one or more bioptic samples are taken from the lesion; and (2) excisional, in which the entire lesion (maximum of 1 cm) is removed. The instruments required to perform an oral biopsy are very common: a dental syringe, vial of local anesthetic, a short needle, silk or synthetic sutures or adhesive containing cyanoacrylate (for chemical suturing), a number 15 or 11 cold knife (used for the fixed oral mucosa of the hard palate, gum, and the labial mucosa) or a biopsy punch of differing diameters (useful for the oral mucosa of the cheeks and tongue), surgical scissors, a klemmer, anatomical and surgical tweezers, and sterile gauze.

An incisional biopsy (similar to that deployed in the two studies reviewed in this manuscript) usually lasts 15 min. Various technical details of performing an oral mucosa biopsy are shown in Fig. 1. After a superficial anesthesia with lidocaine cream, a local anesthesia (Xylocaine® 2%, adrenaline 1:100.000) is performed. The biopsy is performed by an incision with a cold knife (no. 15 or 11) or by punching (usually a 6-mm diameter punch biopsy). When a biopsy punch is performed, the saw blade of this knife is inserted into the mucosa, rotating in a clockwise direction until approximately half its height. The carrot-shaped tissue obtained is cut at its base by a no. 5 cold knife. The surgical wound will be sutured with silk suture or by applying an adhesive containing cyanoacrylate (Fig. 1).

## DEMONSTRATION OF THE ORAL MUCOSA PRODUCTION OF EMA AND ANTI-tTG ANTIBODIES IN PATIENTS WITH CD

There is considerable evidence that the oral mucosa is involved in patients with CD: (1) the demonstration that the oral cavity could be affected by various oral ailments as possible atypical aspects of CD[26,51,52]; (2) the presence of inflammatory changes after oral supramucosal application and a submucosal

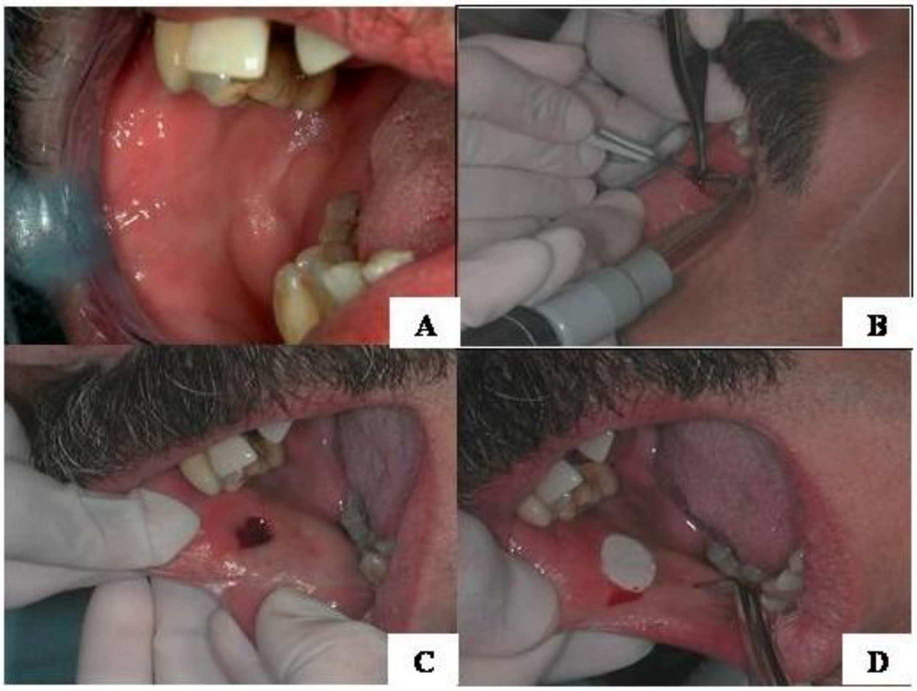

**FIGURE 1.** Oral mucosa biopsy. (A) The specimens were taken from the mouth at the second molar tooth region beneath the occlusal line. (B,C) The biopsies were performed by incision with cold knife or by punching (6-mm diameter punch biopsy). (D) The suture was obtained via the application of an adhesive containing cyanoacrylate.

injection of gliadin in the oral mucosa of CD patients[41,42]; and (3) the close bond between the oral mucosa and immunogenic defense systems.

Generally, this evidence has led two directions of research[49,50] in demonstrating for the first time that the oral mucosa of untreated patients with CD is able to produce CD autoantibodies in *in vitro* culture systems. Patients with signs and/or symptoms compatible with the diagnosis of CD were enrolled in both these studies. All patients were serologically screened for total IgA to exclude IgA deficiency, IgA EMA, and IgA anti-tTG antibodies, and they underwent esophago-gastro-duodenoscopy (EGDS) with duodenal histology examination. The diagnosis of CD in both groups was based on serum EMA and anti-tTG positivity, associated with evidence of the findings of typical CD histopathological abnormalities of small intestinal mucosa, a subsequent disappearance of clinical symptoms, and a negative EMA and anti-tTG after a GFD. The patients with a negative serology and histology for CD were enrolled as controls.

The test group in the study performed by Carroccio et al.[49] included 28 patients with CD (15 adults and 13 children), while the control group contained 14 adult patients. In the study performed by Vetrano et al.[50], 16 and 11 adults comprised the test and control groups, respectively. In both studies, all patients underwent not only a small intestinal biopsy by means of EGDS, but also an oral mucosa biopsy from the cheek. Six duodenal samples and one biopsy sample were obtained for each patient in the study by Carroccio et al.[49]. The oral sample was divided into four parts: four intestinal samples and two oral specimens underwent routine histology evaluation, and two duodenal and two oral specimens were cultured. Of these latter two, one was in the presence and one in the absence of the 31-43 gliadin peptide to assess EMA and anti-tTG production in the culture supernatants.

Four duodenal and two oral biopsies for each patient were performed by Vetrano et al.[50]. Two intestinal specimens underwent routine histological examination, while the remaining two intestinal specimens and the two oral biopsies were cultured to assess CD antibodies. IgA deficiency was not detected in any patients and in one patient enrolled in the study performed by Carroccio and Vetrano, respectively. In the former study[49], the results of the IgA EMA and anti-tTG assayed on the duodenal and oral mucosa culture were identical and remained unchanged when the 31-43 peptide was added to the culture medium. An EMA assayed on the duodenal mucosa culture medium yielded results that were completely concordant with the final diagnoses: they were positive for all CD patients and negative for all patients not suffering from CD. The EMA assayed on the oral mucosa culture medium was positive in 15 of the 28 CD patients (53.6%). Moreover, anti-tTG antibodies assayed on the duodenal mucosa culture medium were positive in all CD patients and negative in all patients not suffering from CD. Anti-tTG antibodies assayed on the oral mucosa culture medium were positive in 16 of the 28 CD patients (57%). None of controls were positive for the intestinal and oral mucosa tests.

The sensitivity of the EMA test on the oral mucosa media was 53.6%, specificity was 100%, and the diagnostic accuracy was 79%. Sensitivity, specificity, and diagnostic accuracy were 57, 100, and 79%, respectively, for the anti-tTG test on oral mucosa. Significantly more severe inflammation, in terms of lymphocytic infiltrate, of the oral mucosa was observed in the patients with CD who proved to be positive for EMA or anti-tTG in the oral mucosa culture medium. This aspect is similar to the well-known observation that there is a positive correlation between the severity of intestinal histology and the presence of serum EMA in CD[53,54].

The study by Vetrano et al.[50] revealed a positive EMA and anti-tTG assayed on the culture media of intestinal mucosa samples in 14/16 and 15/16 CD patients, respectively. Specifically, one patient was negative for EMA and anti-tTG due to a selective IgA deficiency, while the negative IgA EMA in the intestinal culture media exhibited by the second CD patient was explained by the authors[50] to be linked to the small size of the biopsy samples. The authors demonstrated IgA EMA and anti-tTG in all but one of the cultured oral mucosa biopsies from 16 subjects with untreated CD. The single individual whose oral biopsy was negative for both IgA EMA and anti-tTG was characterized by a selective IgA deficiency. Neither EMA nor anti-tTG antibodies were found in sera and in the culture media of the intestinal and oral biopsy samples of the 11 controls. The sensitivity for the anti-tTG test on the oral mucosa demonstrated by Vetrano et al.[50] was 100%, in contrast to the 57% described by Carroccio et al.[49].

In terms of sensitivity and diagnostic accuracy, the different results obtained in these two studies could be linked to the different biopsy size used by the two research groups[55]. Whereas Carroccio et al.[49] used biopsy specimens with an approximate weight of 5 mg, Vetrano et al.[50] highlighted that the use of bioptic samples smaller than 10 mg could be responsible for false-negative results. Due to these discrepancies, various standardized procedures must be performed prior to routinely using the oral mucosa as an area for diagnostic purposes.

Finally, we would like to underline that neither of the two examined studies included patients with CD or with suspected CD on a GFD. Future studies are required to demonstrate whether oral biopsy samples obtained from patients with CD on a GDF and cultured with gliadin peptides will be able to produce CD antibodies or to demonstrate histomorphological modifications. It is the opinion of the authors of this paper that the thesis relating to the immunological reactivity of the oral mucosa and its possible diagnostic potentiality in patients with CD will thus be strengthened.

## IMPLICATIONS AND FUTURE PERSPECTIVES

Although the small intestinal mucosa is the main site of the gut involved in CD, it is known that other mucosal surfaces belonging to the gastrointestinal tract and the GALT can also be involved. Indeed, it has become evident that other parts of the gastrointestinal tract, including oral[25,26,48], esophageal[27,39], gastric[27,28], terminal ileal[29], and rectal mucosa[30,31,33,34,35,40], could be sensitive to gluten ingestion and thus reveal clinical and histological modifications. This has also been demonstrated in atypical forms of CD[26,56], and the rectal mucosa has also been used for gluten challenges[34,36,37,38].

All these data demonstrate that gluten-driven T-cell activation is not restricted to the proximal part of the intestine, but it is present throughout the entire intestine.

Despite these considerations, an examination of the intestinal mucosa is still considered the cornerstone for a definitive CD diagnosis[57]. However, a small bowel biopsy is an invasive procedure that many patients find hard to accept, especially if asymptomatic or only suffering from mild symptoms. In this respect, the research of an easily accessible site for obtaining biopsy samples (for diagnostic and/or research purposes) is most welcome, as the mouth is a much easier and less invasive site to study than the other gastrointestinal mucosal surfaces.

The aforementioned studies, which have demonstrated that the oral mucosa of CD patients is able to produce EMA and anti-tTG in a culture system, open up new and interesting perspectives of study. In both studies, the most encouraging results were 100% specific without the risk of false-positives. As previously mentioned, any discrepancies regarding the sensitivity of the method could be linked to the different biopsy sizes used by the two research groups. The search for easier diagnostic methods other than the small intestine biopsy is very important, above all in patients for whom EGDS is contraindicated. To date, discrepancies regarding the sensitivity obtained from the two studies do not yet make them suitable for routine diagnostic purposes, and further consideration is merited on larger sample sizes with standardized collection and analysis methods.

For the first time, data from these studies have demonstrated that the oral mucosa (like the intestinal mucosa) of patients with a new CD diagnosis, who are still on a gluten diet, was able to produce EMA and anti-tTG in the culture medium, with or without *in vitro* gliadin stimulation. These findings are not surprising since the oral cavity is closely bound to immunogenic defense systems. Although, from an immunological point of view, the structure of healthy oral mucosa differs from that of the bowel, the lymphoepithelial structures of Waldeyer’s pharyngeal ring in the posterior part of the oral cavity, including nasal-associated lymphoid tissues (NALT), constitute an important part of GALT. GALT is the largest and most important part of mucous-associated lymphoid tissue (MALT)[58]. Subsequent to antigen activation and the proliferation and partial differentiation in GALT, memory B and T cells migrate to regional lymph nodes and secretory glands, including salivary glands, where they can begin to produce sIgA[59]. These are very probably the physiological mechanisms that permit the oral mucosa to participate in the immune reaction that characterizes the pathogenesis of CD. However, it must also be underlined that there is now compelling evidence that supports the compartmentalization of the mucosal immune system[60,61]. Activated immune cells, therefore, seem to home preferentially to the sites where they were originally primed[61]. Within this viewpoint, NALT and bronchus-associated lymphoid tissue (BALT) may be more important than GALT in inducing an immune response in the upper aerodigestive tract, including the oral cavity[25].

Further studies should also investigate whether oral gluten-sensitized lymphocytes originate exclusively from the homing of cells primed in GALT or, rather, whether they could also be primed directly in the oral cavity. The latter would have important implications not only for diagnostics, but also for the understanding of CD in general. The hypothesis that gluten could stimulate an immunological response directly in the oral cavity would have important implications for our understanding of the pathogenesis, diagnosis, and management of CD. For example, the hypothesis of an induction of a gluten-related immune response even in the oral cavity should be taken into account in the development of new therapeutic strategies that aim to prevent the triggering of such immune reactions. Of the new, nondietary therapies being studied, the most attractive seems to be the use of enzymes that digest immunogenic gluten peptides[62,63]. Should these peptides be able to elicit an immune response in the oral cavity, enzymatic therapies could be ineffective or not fully effective unless they are already active in the mouth, and not only in the stomach and/or in the small intestine. Moreover, the hypothesis that a gluten-related immune response could be induced in the oral cavity should be taken into account in the development of new nondietary therapies that aim to prevent the triggering of such immune reactions only on the gastrointestinal level. This involves, for instance, the case of drugs that act on intestinal permeability, in particular, on the mechanisms regulated by zolulin family proteins.

In conclusion, the studies performed by Carroccio et al.[49] and Vetrano et al.[50] have demonstrated for the first time that the oral mucosa contributes to the production of EMA and anti-tTG in untreated patients with CD and, consequently, it plays a role in the pathogenesis of the disease. These new data open up new perspectives regarding a diagnosis of CD and for the understanding of the pathogenesis of CD. The oral mucosa could be a simple means with which to study alternative treatments for CD.
